# Supplementary material for: Self-consistent hardness measurements spanning eleven decades of strain rate on a single material surface
Source: Nat Commun. 2025 Jul 4;16:6148. doi: 10.1038/s41467-025-61445-2 (PMC12229530; doi:10.1038/s41467-025-61445-2)
Supplement: Supplementary file 1 — Supplementary Information [file 41467_2025_61445_MOESM1_ESM.pdf]

Supplementary Materials for

Self-consistent hardness measurements spanning eleven decades of strain rate on a single material surface

Authors: Luciano Borasi[1], Christopher A. Schuh[1*]

Affiliations:

[1]Department of Materials Science and Engineering, Northwestern University; Evanston, IL 60208, USA.

*Corresponding author. Email: schuh@northwestern.edu

Comparison of strain rate and hardness data from polished diamonds and microfabricated impactors

Laser-diced polished diamonds (Fig.3A-right of the main manuscript) were launched at velocities comparable to microfabricated impactors (Fig. 3E of the main manuscript), as observed in Figure S1-right. Data points generated using these two different indenter geometries align in terms of computed strain rate and resulting hardness within this regime (Figure S1-left). The inclusion of the microfabricated diamonds does not expand that range of strain rates, per se, because they are also employed at $\sim 10^6$ s^{-1} and are included in this study to show that: (i) diamond indenters can be microfabricated using conventional cleanroom techniques, and (ii) our testing technique is not limited to a single geometry tip, and in fact two different tip shapes give the same result.

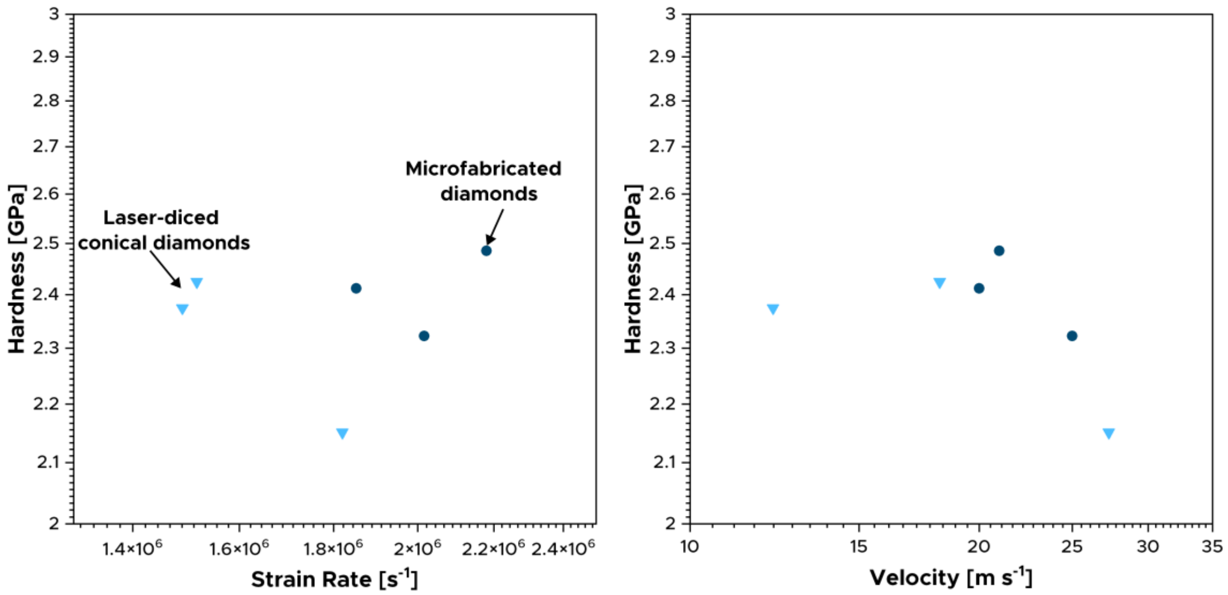

Figure S1 Comparison of strain rate and hardness values in the nickel sample (left) for laser-diced polished diamonds and microfabricated impactors launched at similar velocities (right). Source data are provided as a Source Data file.

Comparison of strain rate and hardness data from polished pyramidal diamonds and a spherical indenter, and from spherical and irregular (sharp) alumina particles.

To further assess the validity of the strain rate definition used in this study, we conducted two additional impact tests, aiming to compare spherical and sharp indenters.

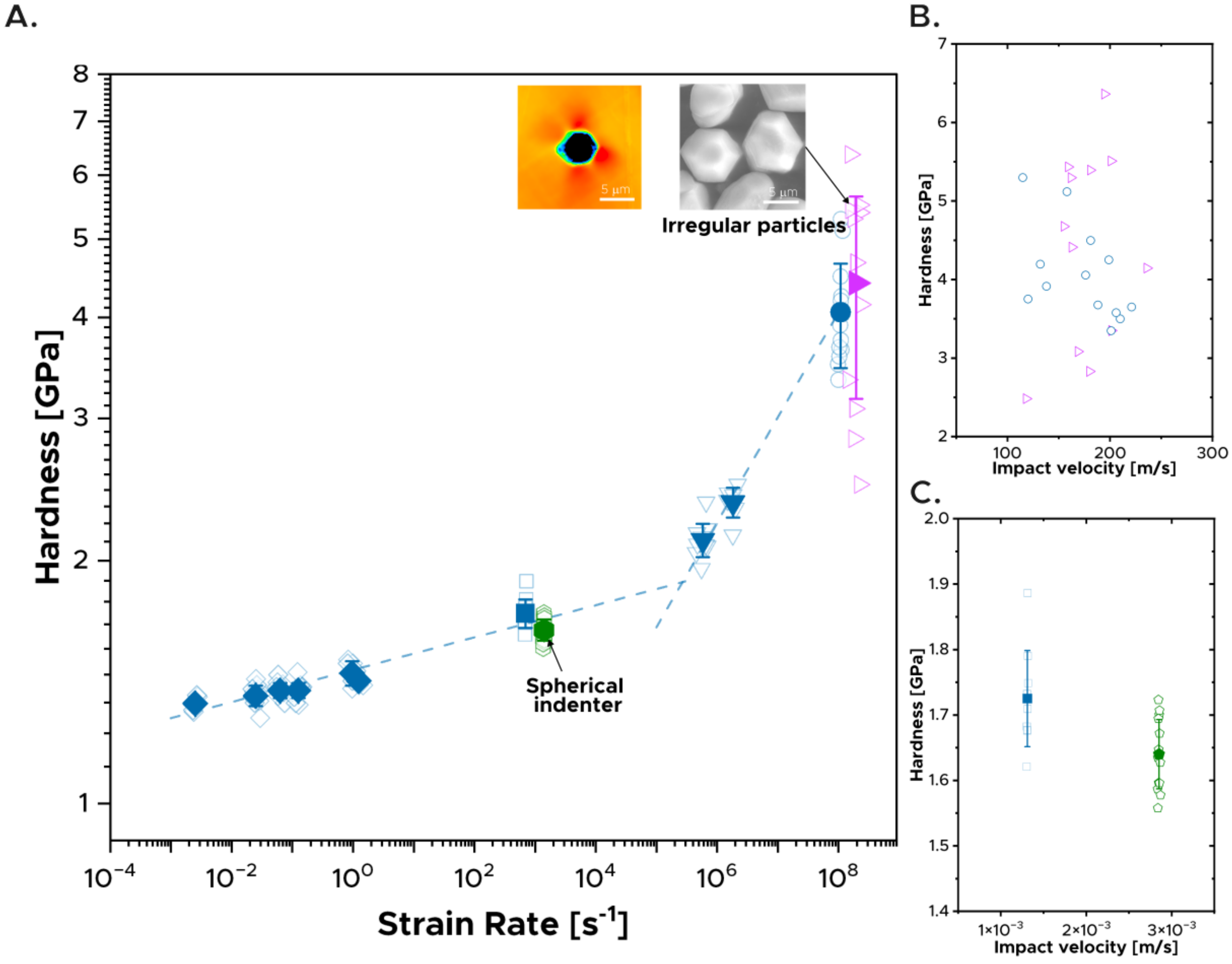

Figure S2 (A) Hardness as a function of strain rate for pure 99.99% nickel containing data acquired by impact indentation employing a spherical indenter (green) and irregular particles launched by conventional LIPIT (purple). Error bars represent the standard deviation of the data. The figure includes a confocal image of an indent left by irregular particles, alongside an SEM image of the particles for reference. (B) Comparison of hardness values for spherical (○-blue) and irregular (▷-purple) alumina particles, impacting at similar velocities. (C) Comparison of hardness values for polished conical diamonds (□-blue) and spherical (○-green) indenter, impacting at similar velocities. Source data are provided as a Source Data file.

(i) Comparison of spherical (D=25 μm) and polished diamond indenters: impact indentation tests were conducted using the NanoTest Alpha instrumented nanoindenter from Micro Materials Ltd. (UK) using a polished pyramidal diamond tip, same as those used in low-strain rate LIPIT, and a spherical diamond tip. The results are compared in Figure S2 in terms of average strain rate (~10^3 s^{-1}), resulting hardness (Figure S2-A) and impact velocity (Figure S2-C). As shown, the computed strain rate and hardness values for the two geometries are in good agreement.

(ii) Comparison of spherical and sharp indentations via LIPIT: Additional LIPIT tests were performed using irregular alumina particles (Sumicorundum - Sumito Chemical Co. LTd,

Japan) (Figure S2-A&B). These irregular particles, observed in Figure S2, produced impact profiles closer to those of sharp indenters (see confocal image in figure S2). Despite a higher spread in hardness values – likely due to uncertainties in estimating impactor mass, as their volume was approximated from side measurements – the computed strain rates remain comparable.

Overall, these additional tests confirm that for both spherical and sharp indentations at about the same impact velocities, the strain rate and the hardness values that result are within uncertainty of one another. The computed strain rate and hardness values thus remain consistent across different impactor geometries, reinforcing the validity of the strain rate definition adopted from conventional nanoindentation. Any discrepancies introduced by shape differences do not affect the main conclusions of this study.

Mass to velocity relationship in LIPIT experiments

Veysset et al. [1] evaluated the maximum velocities achievable with LIPIT launches across a diverse range of masses in under both atmospheric and vacuum conditions. They identified a power law relationship between maximum particle velocity and mass, which we further extend in Figure S3; the purple triangles and blue stars in this figure represent new data from the present work. It is important to note that, as described in [2], launch pads and LIPIT setups have been improved over the years. Nevertheless, Figure S3 demonstrates that this empirical power law continues to represent experimental observation with relatively good accuracy even when the impactor mass is increased by nearly four orders of magnitude. This relationship serves as an invaluable tool for estimating maximum velocity and facilitates the design of LIPIT experiment in advance.

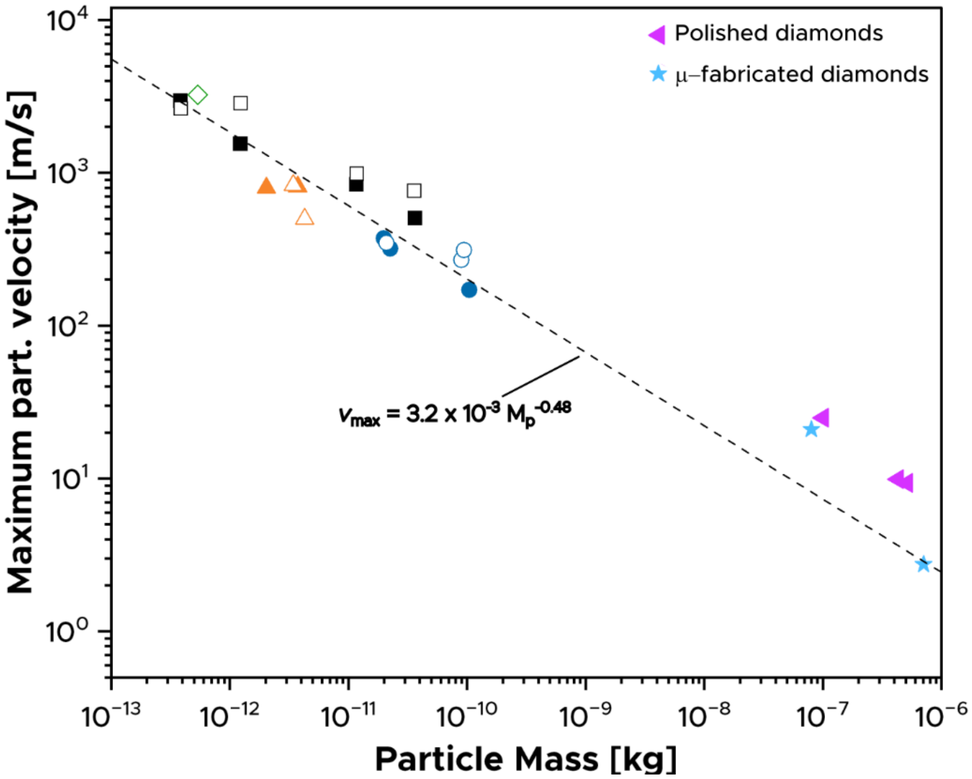

Figure S3 Maximum particle velocity as a function of particle mass for a wide range of particle types (see [1] for details), plotted alongside data for diamond impactors. Source data are provided as a Source Data file.

Microfabrication details

The process flow can be found in [3]. Microfabrication steps were conducted at both NUFAB (Northwestern University, US) and Pritzker Nanofab (University of Chicago, US). The synthetic diamond (7x7x0.3 mm^3, 5-10 nm Ra finish, Evolve Diamonds LLC, US) was first cleaned using Piranha solution (H2SO4(96%):H2O2(30%) (3:1)) for 3 minutes. The plate was exposed to oxygen plasma (100W, 20 sccm O_2, 0.2 mbar; Samco PC300, Japan) for 2 minutes. Immediately following oxygen plasma, an approximately 2 μm thick silicon dioxide layer was deposited by plasma-enhanced chemical vapor deposition (PECVD) (STS LpX CVD, UK). The diamond plate was then attached to a silicon wafer using double sided Kapton tape and an approximately 8 μm thick AZ P4620 photoresist was spin coated at 4000 rpm for 40 seconds, followed by a 3 minute softbake at 110 °C. The exposure of the photoresist was conducted using maskless aligner-Heidelberg MLA150 (405 nm, 1000 mJ cm-2; Heidelberg Instruments, Germany), followed by a development in a pre-diluted AZ 400K 1:4 developer for 160 seconds. The silicon dioxide was etched for 60 minutes using an Apex SLR ICP RIE from PlasmaTherm (500 W ICP power, 50 W bias power, 10 sccm Ar, 5 sccm He, 5 sccm CF_4, 50

sccm CHF_3). The same equipment was used to etch through the diamond substrate for approximately 3 hours in an O_2 based plasma (700 W ICP power, 100 W bias power, 10 mtorr, 30 sccm O_2; etching rate was ~140-160 nm/min). Finally, squares of around 400x400 μm^2 were laser diced around each of the pillars by Evolve Diamonds LLC.

[1] D. Veysset, Y. Sun, S.E. Kooi, J. Lem, K.A. Nelson, Laser-driven high-velocity microparticle launcher in atmosphere and under vacuum, International Journal of Impact Engineering 137 (2020) 103465. https://doi.org/10.1016/j.ijimpeng.2019.103465.

[2] A. Reiser, C.A. Schuh, Microparticle Impact Testing at High Precision, Higher Temperatures, and with Lithographically Patterned Projectiles, Small Methods n/a (n.d.) 2201028. https://doi.org/10.1002/smtd.202201028.

[3] A. Toros, M. Kiss, T. Graziosi, H. Sattari, P. Gallo, N. Quack, Precision micro-mechanical components in single crystal diamond by deep reactive ion etching, Microsyst Nanoeng 4 (2018) 1–8. https://doi.org/10.1038/s41378-018-0014-5.
